# Prevalence of Chronic Kidney Disease in People Living With HIV Following in Dammam Medical Complex, Saudi Arabia

**DOI:** 10.7759/cureus.51947

**Published:** 2024-01-09

**Authors:** Ali Alsaeed, Mousa J Alhaddad, Ridha H Alkhalifah, Faisal A Abu Shaigah, Manal M Alshehab, Zahra H Alali, Sara H Ebrahim, Hasan M Abdulla, Ghadeer A Al Ibraheem, Ghadeer A Al Bensaad, Welaa A Alaliw, Hawra J Alsheef, Mohammed Y Altriki, Abdullah A Alkhalaf

**Affiliations:** 1 Infectious Disease, Dammam Medical Complex, Dammam, SAU; 2 Internal Medicine, Dammam Medical Complex, Dammam, SAU; 3 Nephrology, King Fahad Medical City, Riyadh, SAU; 4 College of Medicine, Mansoura University, Mansoura, EGY; 5 College of Medicine, King Faisal University, Al Hofuf, SAU; 6 College of Medicine, Imam Abdulrahman Bin Faisal University, Dammam, SAU

**Keywords:** saudi arabia, proteinuria, albuminuria, hiv associated nephropathy, hiv, chronic kidney disease (ckd)

## Abstract

Backgrounds

People living with human immunodeficiency virus (HIV) are at a greater risk of chronic kidney disease (CKD) compared to people not having HIV. Evaluating the prevalence of CKD in people living with HIV in Dammam, Saudi Arabia was the main objective of this study.

Methods

This cross-sectional study included adult HIV patients who were followed at Dammam Medical Complex. The patients' demographic data, comorbid conditions, and HIV history were reviewed from their electronic medical records.

Results

A total of 729 patients were counted. The glomerular filtration rate (GFR) of 235 patients could not be estimated. The data for the remaining 494 patients were analyzed. The cohort consisted of 406 male patients (82.19%) and 88 female patients (17.81%). The mean ± standard deviation for the patients' age and HIV duration were 39.08±10.93 years and 4.37±3.15 years, respectively. Ten patients (2.02%) had a GFR of <60 mL/min/1.73 m^2^. Among 136 patients who had an estimated GFR of ≥60 mL/min/1.73 m^2^ and were tested by a urine examination, 27 patients (19.85%) had albuminuria. Combining the two figures resulted in an estimated prevalence of CKD in HIV patients of 21.47%. Only one patient (0.02%) was receiving dialysis.

Conclusions

The prevalence of CKD in people living with HIV in Dammam, Saudi Arabia was higher than the general population. The findings highlight the elevated risk of CKD among people living with HIV and emphasize the importance of regular monitoring and early detection of kidney dysfunction in this population.

## Introduction

With the widespread use of antiretroviral drugs, people living with human immunodeficiency virus (HIV) have a better life expectancy and live longer. However, they have fewer comorbidities-free years compared to people not having HIV [1,2]. Chronic kidney disease (CKD) is considered one of the common comorbidities in people living with HIV [2]. The risk of developing CKD in this population is influenced by several factors, which include low CD4 count, high viral load, hepatitis B and C coinfections, diabetes mellitus, hypertension, and age [3-5]. HIV-associated nephropathy (HIVAN) with a histopathological picture of focal segmental glomerulosclerosis represents the major underlying cause of CKD in this population. However, other causes include drug-induced kidney injury, and complex-mediated kidney disease [3,6,7].

Based on a meta-analysis published in 2018, the overall prevalence of CKD in people with HIV is around 4.8% [8]. Evaluating the prevalence of CKD in people living with HIV in Dammam, Saudi Arabia, was the main objective of this study.

## Materials and methods

This was a cross-sectional chart review study. The inclusion criteria were adult HIV patients who were followed at Dammam Medical Complex, Dammam, Saudi Arabia, in the first half of 2023. The exclusion criteria were patients with no recorded creatinine measurements to allow for estimating their glomerular filtration rate (GFR). Patients' demographic data, comorbid conditions, and HIV history were reviewed from their electronic medical records. The records were also reviewed for the results of their creatinine level, CD4 count, and HIV viral load. The Institutional Review Board of Dammam Medical Complex approved and monitored this study (approval number: IM-12, dated July 17, 2023).

The GFR for the patients was estimated using the 2021 CKD-Epidemiology Collaboration (CKD-EPI) equation [9]. CKD was defined by the presence of decreased kidney function (identified by an estimated GFR of less than 60 mL/minute per 1.73 m^2^) or markers for kidney damage for three or more months, irrespective of the cause [10]. The most recent creatinine value for each patient was used to estimate the GFR. To ensure that the needed duration was fulfilled, the most recent creatinine value was reviewed in conjunction and comparison with the preceding two creatinine values. The patients with CKD were staged using the Kidney Disease Outcomes Quality Initiative (KDOQI) classification [10]. 

The CKD prevalence was calculated as: (the actual number of patients with GFR of <60 mL/minute/1.73 m^2^ + the estimated number of patients with GFR of ≥60 mL/minute/1.73 m^2^ and albuminuria)/(the number of all patients).

The patients with normal GFR were compared with the patients with decreased GFR (including those with mildly decreased GFR between 60 and 89 mL/minute/1.73 m^2^) looking for any association between impaired kidney function and patients' demographics, comorbid conditions, and personal HIV data. 

The Python programming language version 3.7.6 (Python Software Foundation, Wilmington, Delaware, United States) with the use of the SciPy library 1.4.1 (Enthought, Inc., Austin, Texas, United States), and Statsmodels module (v0.11.1, Python package) was used to analyze the patients' data. Descriptive statistics (i.e., mean, standard deviation, median, interquartile range, count, and percentage) were provided as necessary. The normality of the data was tested using the Shapiro-Wilk Test. The Chi-square test was used to compare the categorical variables, and the two-sample t-test was used to compare the continuous variables. The Wilcoxon rank-sum test was used to compare non-normal distributed continuous variables. A p-value < 0.05 was assumed to mark statistical significance.

## Results

A total of 729 patients were counted. The GFR of 235 patients could not be estimated. The data for the remaining 494 patients were analyzed. The cohort consisted of 406 male patients (82.19%) and 88 female patients (17.81%), with a male-to-female ratio of 4.61. The median (interquartile range (IQR)) for the patients' age and HIV duration were 36.52 (31.44-44.49) years and 3.63 (1.85-6.25) years, respectively. The median viral RNA load was 20 copies/mL with an IQR of 0-112.5 copies/mL. The median CD4 count was 660 cells/microL with an IQR of 393.6-945.5 cells/microL. Four hundred and forty-eight patients (90.69%) were receiving HIV medications; 43 patients (8.7%) were diabetic and 23 patients (4.66%) were hypertensive. The patients' demographics are shown in Table 1.

**Table 1:** Patient Demographics (n = 494)

Characteristic			Values/Frequency
Age (years), mean ± SD			39.08±10.93
Gender	Male, n (%)	406 (82.19%)	
	Female, n (%)	88 (17.81%)	
HIV Duration (years), mean ± SD			4.37±3.15
CD4 Count (cells/microL), mean ± SD			717.06±480.42
HIV Medications	Bictegravir, Emtricitabine, and Tenofovir Alafenamide, n (%)	236 (47.77%)	
	Dolutegravir, Emtricitabine and Tenofovir Alafenamide, n (%)	85 (17.21%)	
	Darunavir Cobicistat, Emtricitabine and Tenofovir alafenamide, n (%)	46 (9.31%)	
	Raltegravir, Emtricitabine and Tenofovir Alafenamide, n (%)	41 (8.3%)	
	Abacavir, Dolutegravir, and Lamivudine, n (%)	31 (6.28%)	
	Others, n (%)	9 (1.82%)	
	None, n (%)	46 (9.31%)	
Hypertension, n (%)			23 (4.66%)
Diabetes Mellitus, n (%)			43 (8.7%)

The mean (IQR) for creatinine level and estimated GFR were 0.90 (0.76-1.03) mg/dL and 106.69 (92.81-116.85) mL/min/1.73 m^2^, respectively. Three hundred and seventy-seven patients (76.32%) had a normal estimated GFR (≥90 mL/min/1.73 m^2^) and 107 patients (21.66%) had a mildly decreased estimated GFR (60-89 mL/min/1.73 m^2^). The remaining 10 patients (2.02%) had a GFR in the CKD range (<60 mL/min/1.73 m^2^) with one of them being on dialysis (0.02%). The patients' GFR stages are shown in Table 2.

**Table 2:** Patients' GFR Stages (n = 494) GFR: glomerular filtration rate

Characteristic		Values/Frequency
Creatinine (mg/dL), mean ± SD		0.93±0.41
Estimated GFR (mL/minute/1.73 m^2^), mean ± SD		103.48±19.86
GFR Stage, n (%)	≥90 mL/minute/1.73 m^2^	377 (76.32%)
	60-89 mL/minute/1.73 m^2^	107 (21.66%)
	30-59 mL/minute/1.73 m^2^	7 (1.42%)
	15-29 mL/minute/1.73 m^2^	0 (0.00%)
	<15 mL/minute/1.73 m^2^	3 (0.60%)

Only 143 patients (28.95%) were tested by a urine examination for albuminuria. Among the patients who had an estimated GFR of ≥60 mL/min/1.73 m^2^ and were tested by a urine examination (n=136), the urine examination was positive for albumin in 27 patients (19.85%). This resulted in an estimated prevalence of CKD in HIV patients of 21.47%.

The patients with GFR ≥90 mL/min/1.73 m^2^ were generally younger than the patients with GFR <90 mL/min/1.73 m^2^ (36.65 vs 46.9 years on average, p-value <0.001). Moreover, they had fewer diabetic patients (6.63% vs 15.38%, p-value <0.01) and hypertensive patients (2.12% vs 12.82%, p-value <0.001). The detailed comparisons between the patients based on their GFR are shown in Table 3. 

**Table 3:** Comparison between Patients with GFR ≥90 mL/minute/1.73 m2 and Patients with GFR <90 mL/minute/1.73 m2 (n = 494) * A p-value of less than 0.05 was used to indicate statistical significance. GFR: glomerular filtration rate

Characteristic	GFR ≥90 mL/minute/1.73 m^2^ (n = 377)	GFR <90 mL/minute/1.73 m^2^ (n = 117)	p-value
Age (years), mean ± SD	36.65±9.08	46.9±12.63	0*
Female, n (%)	69 (18.3%)	19 (16.24%)	0.7105
Diabetes Mellitus, n (%)	25 (6.63%)	18 (15.38%)	0.006
Hemoglobin A1C (%), mean ± SD	5.62±1.25	6.05±1.66	0.0119*
Hypertension, n (%)	8 (2.12%)	15 (12.82%)	0*
HIV Duration (years), mean ± SD	4.28±3.17	4.66±3.1	0.2592
CD4 Count < 200 cells/microL, n (%)	21 (9.95%)	6 (7.89%)	0.7659
On HIV Medications, n (%)	343 (90.98%)	105 (89.74%)	0.8255

## Discussion

The global prevalence of CKD based on the Kidney Disease: Improving Global Outcomes (KDIGO) definition is 13.4% [11], while the prevalence of CKD in the general population in Saudi Arabia is 5.7% with 0.4% of patients having GFR of less than 60 mL/minute/1.73 m^2^ and 5.3% of patients having albuminuria [12]. In the present cohort, the prevalence of CKD was 21.5% with 2% of patients having a GFR below 60 mL/minute/1.73 m^2^. This higher prevalence of CKD in people living with HIV than the CKD prevalence in the general population both locally and globally highlights the substantial burden of kidney dysfunction among HIV patients. It should emphasize the significance of regular monitoring of kidney function in this population, especially since our findings were in line with the previous reports that evaluated the prevalence of CKD in HIV patients. A study that was conducted in China showed that 5.6% of people living with HIV had a GFR below 60 mL/minute/1.73 m^2^ and that 13.7% of the patients had proteinuria with an overall prevalence of CKD of 16.8% [13]. Moreover, a study in Nigeria and another in Italy found a CKD prevalence of around 22% [14,15].

Albuminuria, a marker of kidney damage, was found in 19.85% of those with an estimated GFR ≥60 mL/minute/1.73 m^2^ who underwent a urine examination. This is comparable to the results of a study that was conducted in Riyadh in the central region of Saudi Arabia, which showed that 12% of the tested HIV patients had albuminuria [16]. However, the presence of a high percentage of HIV patients who were not evaluated for albuminuria (71% in the present cohort and 56% in the Riyadh study [16]) points toward a need to raise awareness for testing and quantifying albuminuria in people living with HIV. 

It is noteworthy that a significant proportion of the patients (90.69%) were receiving HIV medications, indicating a high engagement in antiretroviral therapy. However, comorbid conditions were also prevalent within the cohort, with 4.66% of the patients being hypertensive and 8.7% having diabetes. These comorbidities are known to potentially impact kidney health and contribute to the development or progression of kidney disease [4-6]. Therefore, it was not surprising that the prevalence of diabetes mellitus (12.83% versus 2.12%) and hypertension (12.83% versus 2.12%) was significantly higher in those with renal impairment compared with the patients with normal GFR.

The study had several limitations. Firstly, the study had a cross-sectional design. Therefore, neither a casualty nor a temporal relationship between HIV and the development of CKD could be determined. Secondly, the data was collected retrospectively, which can lead to potential biases due to the presence of missing or inaccurate data. This was markedly evident as 235 patients were excluded for not having enough data to determine their GFR. Similarly, most of the remaining included patients had not done a urine examination to evaluate them for albuminuria. Finally, the study was conducted at a single center, which may not represent the entire HIV population. Due to the HIV-related stigma, patients might not seek medical attention or choose to be managed in centers other than Dammam Medical Center, a governmental hospital. Such patients were not represented.

## Conclusions

The high prevalence of CKD in people living with HIV emphasizes the importance of regular monitoring and early detection of kidney dysfunction in this population. Future research should aim to address the limitations of our study by conducting prospective studies with larger cohorts. Such studies would increase our understanding of the progression of CKD over time in people living with HIV and the effects of antiretroviral therapy and viral suppression. Exploring interventions and strategies for early detection and management of CKD in this population could help improve outcomes and quality of life for individuals living with HIV.
